# *Pithecellobium clypearia*: Amelioration Effect on Imiquimod-Induced Psoriasis in Mice Based on a Tissue Metabonomic Analysis

**DOI:** 10.3389/fphar.2021.748772

**Published:** 2021-09-17

**Authors:** Ying Li, Jiaxin Zong, Wenjun Ye, Yuanfeng Fu, Xinyi Gu, Weisong Pan, Li Yang, Ting Zhang, Mingmei Zhou

**Affiliations:** ^1^ Institute for Interdisciplinary Medicine Sciences, Shanghai University of Traditional Chinese Medicine, Shanghai, China; ^2^ School of Pharmacy, Shanghai University of Traditional Chinese Medicine, Shanghai, China; ^3^ Wuhan Institute for Drug and Medical Device Control, Hubei, China

**Keywords:** *Pithecellobium clypearia* Benth. (*Archidendron clypearia* (Jack) I.C.Nielsen), imiquimod, psoriasis, metabolomics, mechanisms

## Abstract

*Pithecellobium clypearia* Benth. (accepted name: *Archidendron clypearia* (Jack) I.C.Nielsen; Mimosaceae), a popular traditional Chinese medicine, has a significant anti-inflammatory effect. The crude water extract of the aerial part of *P. clypearia* has been clinically applied to treat upper respiratory tract infections, acute gastroenteritis, laryngitis, and pharyngitis. However, the therapeutic mechanism of ethanol fraction of water extract (ESW) of *P. clypearia* to treat psoriasis should be complemented. The aim of our research was to clarify the protective effects of ESW from *P. clypearia* against psoriasis-like skin inflammation induced by imiquimod (IMQ) in mice with efficacy indexes and target tissue (spleen and serum) metabolomics. The ingredient of ESW was analyzed by ultrahigh-performance liquid chromatography combined with tandem mass spectrometry (UHPLC-MS/MS) method. The imiquimod-induced psoriatic mouse model was employed to investigate the effect of ESW against psoriasis, where the treatment method was implemented for 6 days both topically (Gel at 5%) and orally (at 2.4 g/kg p.o.). Traditional pharmacodynamic indicators (phenotypic characteristics, psoriasis area and severity index (PASI) score, H&E staining, immunohistochemical staining, the thickness of epidermis, body weight change, and spleen index) were conducted to appraise the efficacy of ESW. Furthermore, a gas chromatography-mass spectrometer (GC-MS) coupled with multivariate analysis was integrated and applied to obtain serum and spleen metabolic profiles for clarifying metabolic regulatory mechanisms of ESW. The current study illustrated that ESW is composed mainly of gallic acid, ethyl gallate, quercitin, 7-O-galloyltricetiflavan, quercetin, and myricetin by UHPLC-MS/MS analysis. ESW could distinctly improve IMQ-induced psoriasis in mouse through reducing PASI score, alleviating tissue damage, restoring spleen index, and inhibiting proliferating cell nuclear antigen (PCNA) expression in psoriasis-like skin tissue. From the metabolomics study, 23 markers with significant changes are involved in eight main pathways in spleen and serum samples, including linoleic acid metabolism and glycine, serine, and threonine metabolism. The current study showed that ESW had obvious antipsoriasis effects on IMQ-induced psoriasis in mice, which might be attributed to regulating the dysfunction of differential biomarkers and related pathways. In summary, ESW of *P. clypearia* showed a favourable therapeutic effect on IMQ-induced psoriasis, and metabolomics provided insights into the mechanisms of ESW to the treatment of psoriasis.

## Introduction

Psoriasis is a chronic inflammatory and immune-mediated skin disease. As a common disease, its incidence accounts for about 2% of the global population, and its prevalence is related to geographical regions (Shrivastava et al., 2015; Ogawa et al., 2018). Psoriasis vulgaris, inverse psoriasis, guttate psoriasis, and pustular psoriasis are typical clinical classification (Rendon and Schäkel, 2019). It presents clinically as sharp erythema, scaly patches, and thickening that may spread to all parts of the body (Parmar et al., 2017). The complicated interplay between innate and adaptive immune systems is associated with potential pathological mechanisms. Notably, various factors were related to the pathogenesis of psoriasis, including genetic factors, the immune system, and environmental conditions. Therefore, psoriasis is usually recognized as a multifactorial disease. Some inflammatory cytokines including tumor necrosis factor-α (TNF-α), interleukin- (IL-) 23, and IL-17 derived from T cells and dendritic cells, macrophages, and keratinocytes play a key role in the pathogenesis of psoriasis (Kamiya et al., 2019). And in some cases, the occurrence of psoriasis can persist even after the treated drug has been not continued. Additionally, a great quantity of data told us the association of psoriasis with multiple comorbidities, such as kidney diseases, angiocardiopathy, therioma, and mood disorders (Takeshita et al., 2017). Although there are some systemic drugs with substantial clinical experience (i.e., acitretin, methotrexate, and cyclosporine), the well-known adverse effects of those drugs, teratogen, hepatotoxicity, and nephrotoxicity, are also still inevitable (Kim et al., 2017).

Discovery of safe and effective antipsoriatic drugs has become an urgent prerequisite for the treatment of psoriasis in theory and practice. Many Traditional Chinese medicines (TCMs) have been recently employed as complementary and alternative medicines of psoriasis based on their original ethnopharmacological uses. Yinxieling tablets which were devised on the basis of TCM theory and were theoretically effective and safe, a Chinese Medicine Compound Preparation with 10 elements, were applied for the treatment of psoriasis (Deng et al., 2017); Total glucosides of paeony alleviated the pathological symptoms of psoriasis-like mice by inhibiting T helper 17 cell differentiation and keratinocytes proliferation (Li et al., 2019). Moreover, a random controlled experiment research about the traditional drug in conjunction with Chinese herbal medicine (YXBCM01) amelioration for psoriasis vulgaris also supplied clinical proof for herbal therapies for psoriasis (Parker et al., 2014).

*Pithecellobium clypearia* Benth. (accepted name: *Archidendron clypearia* (Jack) I.C.Nielsen; Mimosaceae) is widely cultivated in the South of China, such as Guangdong, Yunnan, and Sichuan provinces, and is a prominent and clinically applied traditional Chinese medicine (Li et al., 2016). It is reported that flavonoids are the main active ingredients in *P. clypearia* possessing anti-inflammatory and antiviral properties. In China, TCM doctors use Chinese traditional patent medicine obtained from the extract of aerial part of *P. clypearia* to treat various infections and inflammatory conditions, such as upper respiratory tract infections, laryngitis, pharyngitis, acute gastroenteritis and tonsillitis, and bacterial dysentery (Li et al., 2006; Bao et al., 2009; Li et al., 2016). However, there are no reports on the pharmacological effects and its underlying mechanisms of *P. clypearia* in therapeutic potential of psoriasis.

Metabolomics is considered an integrative method for the comprehensive analysis of all small molecules in a biological system (Chao De La Barca et al., 2015). The identification of diverse metabolites and corresponding metabolic pathways in intricate regulatory mechanisms is achieved through gas chromatography-mass spectrometry (GC-MS), nuclear magnetic resonance (NMR), and liquid chromatography-mass spectrometry (LC-MS) to examine variation in endogenous low molecular weight metabolites (Nicholson and Lindon, 2008). It is worth mentioning that GC-MS has developed as an important and valuable analytical technique in metabolomics research due to its high sensitivity and adequate metabolite resolution (Fiehn, 2016). Furthermore, metabolic profiling based on GC-MS played a dominant role in early diagnosis and pathogenesis of the disease, role mechanism of personalized medicine, and the illumination of complicated biological processes (Hollywood et al., 2015; Kang et al., 2017; Liu et al., 2020). Metabolomics emphasizes a comprehensive explanation of the entire biological system and analysis of the entire compositions rather than analysis of individual metabolites, which is especially in accordance with the comprehensive and integrity personality of TCM (Jia et al., 2013).

In our present study, the therapeutic effects of *P. clypearia* in treating psoriasis-like skin inflammation induced by imiquimod (IMQ) were investigated. Moreover, the efficacy of *P. clypearia* was evaluated by a metabolomics method combining GC-MS with multivariate statistical techniques.

## Materials and Methods

### Chemicals and Reagents

The twigs and leaves of *P. clypearia* were collected from Hutchison Whampoa Guangzhou Baiyunshan Chinese Medicine Co., Ltd. and the voucher specimen (No. 170719) has been deposited in Shanghai University of Traditional Chinese Medicine, Shanghai, China. Heptadecanoic acid was purchased from Aladdin (Shanghai, China). Methoxyamine hydrochloride and N,O-bis(trimethylsilyl)trifluoroacetamide (BSTFA) were obtained from SUPELCO (Bellefonte, PA, United States). Methanol and ethanol were of analytical grade from Shanghai Titan Scientific Co., Ltd. Formic acid was purchased from Shanghai Experiment Reagent Co., Ltd. (Shanghai, China). Acetonitrile (ACN) of HPLC grade was obtained from Merck Company (Darmstadt, Germany). Gallic acid, ethyl gallate, quercetin, myricetin, quercitin, and 7-O-galloyltricetiflavan were isolated from *P. clypearia* by the authors of the present study. Deionized water (18.2 MΩ cm) was prepared using a Milli-Q Direct 8 (Millipore, Bedford, MA, United States).

### Sample Preparation

The air-dried powdered plant material (800 g) was extracted at room temperature with 95% ethanol. The insoluble residue was extracted with dH_2_O four times in 8000 ml water for periods of 2 h each time at 100°C and then extracted with ethanol. The mixture was filtered through filter paper, and the filtrate was evaporated under reduced pressure in a vacuum drying oven (Shanghai Di Po Scientific Instrument Co., Ltd., China). The ethanol fraction of water extract (ESW) was then kept in a desiccator until use.

### Identification of Ingredients of ESW by UPLC-Triple Quad-MS/MS

The phytochemical profiling of ESW was separated and identified by an AB Sciex Triple Quad™ 5500 mass spectrometer (Foster City, CA, United States) equipped with a UHPLC system obtained from Waters (Milford, MA, United States). Chromatographic separation was conducted with ACQUITY UPLC BEH C18 column (2.1 mm × 100 mm, 1.7 μm) (Waters Technologies, United States).

The flow rate of mobile phases consisting of solvent A (0.1% formic acid) and solvent B (acetonitrile) was offset at 0.35 ml/min. The column temperature was set at 35°C and 10 μl sample was injected *via* autosampler. The procedures applied for gradient elution were as follows: 0–5 min, 95–70% A; 5–8 min, 70–20% A; 8–8.1 min, 20–95% A; 8.1–9 min, 95–95% A.

To distinctly identify the analytes, the positive and negative polarity switching mode with multiple reaction monitoring (MRM) were used. The MS with a turbo ion spray (TIS) source was employed to acquire data, and the detailed mass spectrometer conditions were as follows: mass range set as m/z 30–500 for the Triple Quad-MS scan, Collision Gas (CAD) as 7, Curtain Gas (CUR) as 35, Ion Source Gas 1 (GS1) as 50, Ion Source Gas 2 (GS2) as 50, IonSpray Voltage (IS) as −4500 (negative ionization mode), and IS as 4500 (positive ionization mode). The accumulation time for MS/MS scan was kept at 100 ms, resulting in a cycle time of 0.73 s.

### Animal Experiments

Male Balb/c mice weighing 18–22 g were acquired from Shanghai SLAC Laboratory Animal Co., Ltd. (certification NO. SCXK 2018-0006). All animals were maintained in a room where conditions are strictly controlled (temperature 23 ± 3°C, relative humidity 40–70%) under a 12/12 h light-dark cycle and fed with commercially available food and water *ad libitum* all the time. The mice were allowed to acclimatize to the environment for 1 wk prior to experiment. All experimental protocols were performed after approval from Animal Experiment Guides of Shanghai University of Traditional Chinese Medicine.

Twenty-four male Balb/c mice were randomly divided into three groups. The groups were a control group (Con group), a model group (IMQ group), and an ESW treatment group (ESW group). Each group contained eight mice and daily was treated as follows: Con group, mice treated with the vehicle (ddH2O, 400 μl); IMQ group, mice treated with the vehicle and IMQ; ESW group, mice treated with ESW (2.4 g/kg) after IMQ. The mice’s backs were shaved (2 cm × 3 cm) by a depilatory machine and then applied commercially available imiquimod (IMQ) 5% w/w cream (Mingxin Pharmaceuticals, Sichuan, China) at a daily dose of 42 mg on the back skin every day. The whole experiment duration was 7 days.

### Severity Scoring of Skin Inflammation

As an objective scoring system, the clinical psoriasis area and severity index (PASI) was employed to assess the psoriatic lesions ponderance in the back skin (El Malki et al., 2013). Erythema, scaling, and thickness were monitored and scored independently on a scale from 0 to 4: 0 for no avail; 1 for a little influence; 2 for a medium influence; 3 for a serious influence; and 4 for a very serious influence. The score for each group was averaged, and trend lines were drawn to observe the changes in mouse skin lesions. The total score denotes the severity of inflammation.

### Histological Analysis

Mice were sacrificed by cervical dislocation at the end of the seventh day of treatment. The psoriatic skin tissues were removed instantly, then fixed in 4% (w/w) paraformaldehyde solution, and embedded in paraffin wax. Hematoxylin and Eosin (H&E) was used to stain 5 μm sections, and the finished product was observed for pathological analysis by means of a digital microscope (Nikon Eclipse E100, Japan). ImageJ software (National Institutes of Health, Bethesda, MD, United States) was employed to measure epidermal thickness, an accepted mode for evaluating the severity of psoriasis.

### Immunohistochemistry

The sections (5 μm) were dewaxed in xylene and hydrated in graded ethanol solutions (100, 85, and 75%). After antigen retrieval with citrate buffer and being blocked with 3% H_2_O_2_ for 25 min, the sections were incubated with a first antibody (PCNA, 1:500, lot number: GB11010; Servicebio, Wuhan, China) overnight at 4°C, followed by incubation with a second antibody (HRP-goat anti-rabbit antibody, 1:500, lot number: GB23303; Servicebio, Wuhan, China) at room temperature for 30 min. DAB was applied to color development for 5 min and counterstained with hematoxylin for 3 min. Finally, the results of the experiment were observed under a microscope (Nikon Eclipse E100, Japan).

### Sample Collection for Metabolic Analysis

The whole animals were weighed, and the spleen tissues of all animals were removed and weighed immediately and then were stored at a temperature of −80°C in order to develop subsequent experiments. The blood of mice was gathered by the postorbital venous plexus method on day 7. All blood samples were transferred to the Eppendorf tubes, followed by centrifugation at 4000 rpm, 4°C for 10 min after 2 h. The supernatants were transferred to an EP tube and then placed at −80°C until further usage.

Before the analysis, all samples were thawed at 4°C. Samples of spleen tissue were homogenized at an amount of 50 mg with 500 μl water-methanol-chloroform (2:5:2, v/v/v). Serum samples of 50 μl of were mixed with 200 μl methanol-chloroform (3:1, v/v) for protein deposition. The mixture was blended by ultrasonic instrument for 5 min, incubated for 20 min at −20°C, and then centrifuged at 13,000 rpm for 10 min at 4°C to withdraw the solid debris and keep the supernatant. Subsequently, 300 μl clear supernatant and 20 μl 1 mg/ml heptadecanoic acid (internal standard) were mixed to transfer into a GC vial for GC/MSD analysis. The blended solution was concentrated to dryness with N_2_ gas. Additionally, the residues were methoxyximated with 50 μl of 15 mg/ml methoxyamine in pyridine for 1.5 h at 30°C, and then we added 50 μl BSTFA with 1% TMCS (trimethylchlorosilane) and incubated the mixture in a constant temperature drying oven at 70°C for 1 h. In order to test and verify the stability of the established method, the quality control (QC) samples were analyzed together with the experimental samples, which were formed by combining all samples of equal volume and processing according to the same procedure. Each set of eight samples run was followed by one QC sample.

### GC/MSD Analysis

Each 1 μl aliquot of sample was injected into a GC system (Agilent 5975B, GC/MSD system) with a DB-5MS capillary column (30 m × 0.25 mm i.d., 0.25 μm film thickness, 5% diphenyl cross-linked 95% dimethylpolysiloxane; Agilent J&W Scientific, Folsom, CA, United States). Experimental samples were analyzed in a random order. For maintaining stability and reproducibility of the GC-MS systems, three blank replicates and one QC replicate were detected after every eight analytical samples. The carrier gas was helium at a regular flow rate of 1 ml/min and the MS conditions were as follows: the injection temperature was maintained at 260°C, the ion source was set at 230°C, and the quadrupole temperature was adjusted to 150°C. The electron energy was 70 eV. For better resolution, the column temperature was kept at 90°C for 1 min and then programmed at 10°C/min from 90°C to 180°C, increased to 240°C at a rate of 5°C/min, ramped at 25°C/min to 290°C, and held at the final temperature for 11 min during spleen sample processing. The mass spectrometry was performed in the full scan in arrange of 50–450 m/z, and a solvent delay was set for 5 min. The GC oven temperature was initially held at 80°C for 2 min and then increased to 240°C at a rate of 5°C/min. After that, the temperature was raised to 290°C at a rate of 25 C/min and was kept for 10 min during serum sample processing. Mass spectrometry was performed in a full scan with a range of 30–550 m/z, and the solvent delay was set for 7 min.

### Statistical Analysis

NIST software was applied to analyze the mass spectrum information of metabolites. The unprocessed GC/MSD raw files were transformed into NetCDF format *via* Agilent MSD workstation, subsequently filled in missing peaks, and pretreated according to our previous published work (Wang et al., 2012). The result table (TSV file) including charge-to-mass ratio, sample message, and peak area was exported into Microsoft Excel. All data were analyzed after the total spectrum was normalized using multivariate statistical analysis tools through pattern recognition. SIMCA-P 14.1 software (Umetrics, Umea, Sweden) was applied with principal components analysis (PCA), partial least-squares discriminant analysis (PLS-DA), and orthogonal partial least-squares discriminate analysis (OPLS-DA). An S-plot and a permutation test were generated with the identical software. Finally, if the variable importance in the projection value (VIP) of the changed metabolites acquired from OPLS-DA model data was statistically significant (VIP >1) between the Con and IMQ groups or the IMQ and ESW groups, SPSS 21.0 (SPSS, Chicago, United States) was applied to calculate the significant difference (*P*). Pathway analysis of the differential metabolites was confirmed with MetaboAnalyst 3.0 (http://www.metaboanalyst.ca/). Additionally, the statistical analysis for phenotypic characteristic parameters was assessed using Graphpad Prism 5.0 (GraphPad Software Inc., United States). The analytic results were presented as means ± SD. Moreover, differences were considered statistically significant when *P* was below 0.05.

## Results

### LC-MS/MS Method for the Qualitative Analysis of ESW

**FIGURE 1:**
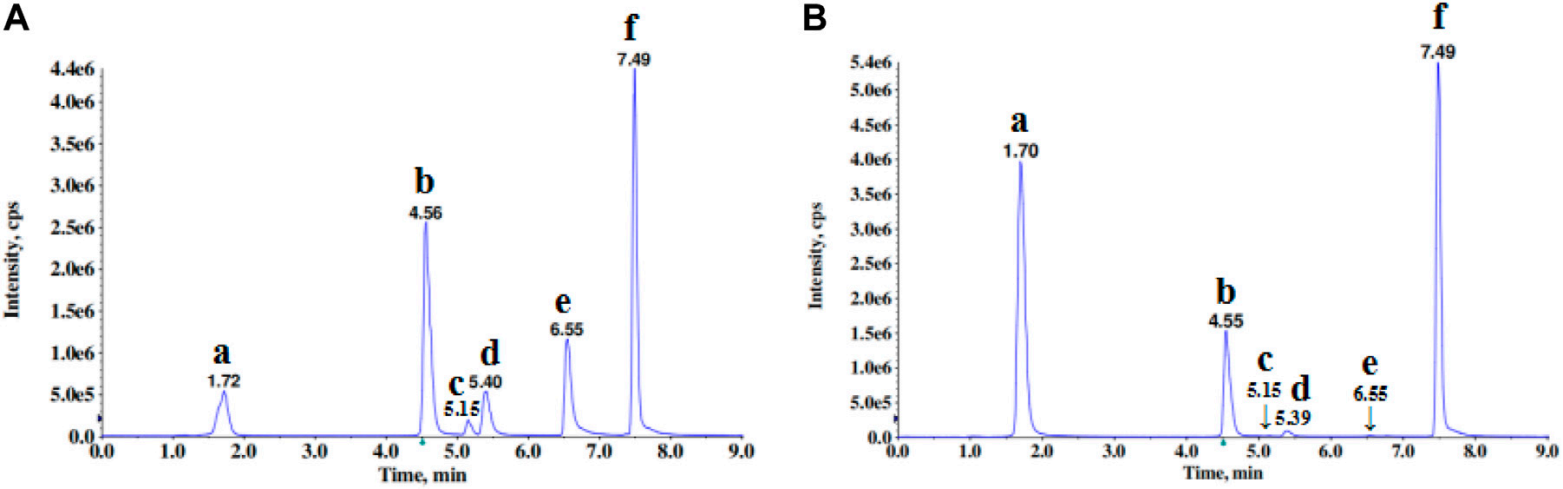
The TICs of the six analytes in mixed standard **(A)** and sample **(B)** by UHPLC-MS/MS in positive and negative ion mode. Alphabets corresponded to the compounds as follows: a, gallic acid; b, ethyl gallate; c, quercitin; d, 7-O-galloyltricetiflavan; e, quercetin; f, myricetin.

The total ion current (TIC) chromatogram of ESW was shown in Figure 1. The major component of ESW was identified by comparing individual peak retention time and molecular weights with that of the reference standard component. The peaks of six components in the TIC chromatogram acquired by LC-MS/MS under MRM mode were characterized, which represented six different chemical constituents, namely gallic acid, ethyl gallate, quercitin, 7-O-galloyltricetiflavan, quercetin, and myricetin (Table 1).

**TABLE 1:** Representative LC/MS/MS data of primary active constituents identified in ESW from *P. clypearia*.

Peak No.	t_R_ (min)	Compound name	Formula	Experimental *m/z*	Fragment ions
a	1.70	Gallic acid	C_7_H_6_O_5_	169[M-H]^−^	125
b	4.55	Ethyl gallate	C_9_H_10_O_5_	197[M-H]^−^	124
c	5.15	Quercitin	C_21_H_20_O_11_	449[M-H]^+^	303
d	5.39	7-O-Galloyltricetiflavan	C_22_H_18_O_10_	441[M-H]^−^	137
e	6.55	Quercetin	C_15_H_10_O_7_	301[M-H]^−^	151
f	7.49	Myricetin	C_15_H_10_O_8_	319[M-H]^+^	256

### Psoriasis-Like Skin Inflammation Induced by IMQ Was Ameliorated by ESW on Mice

**FIGURE 2:**
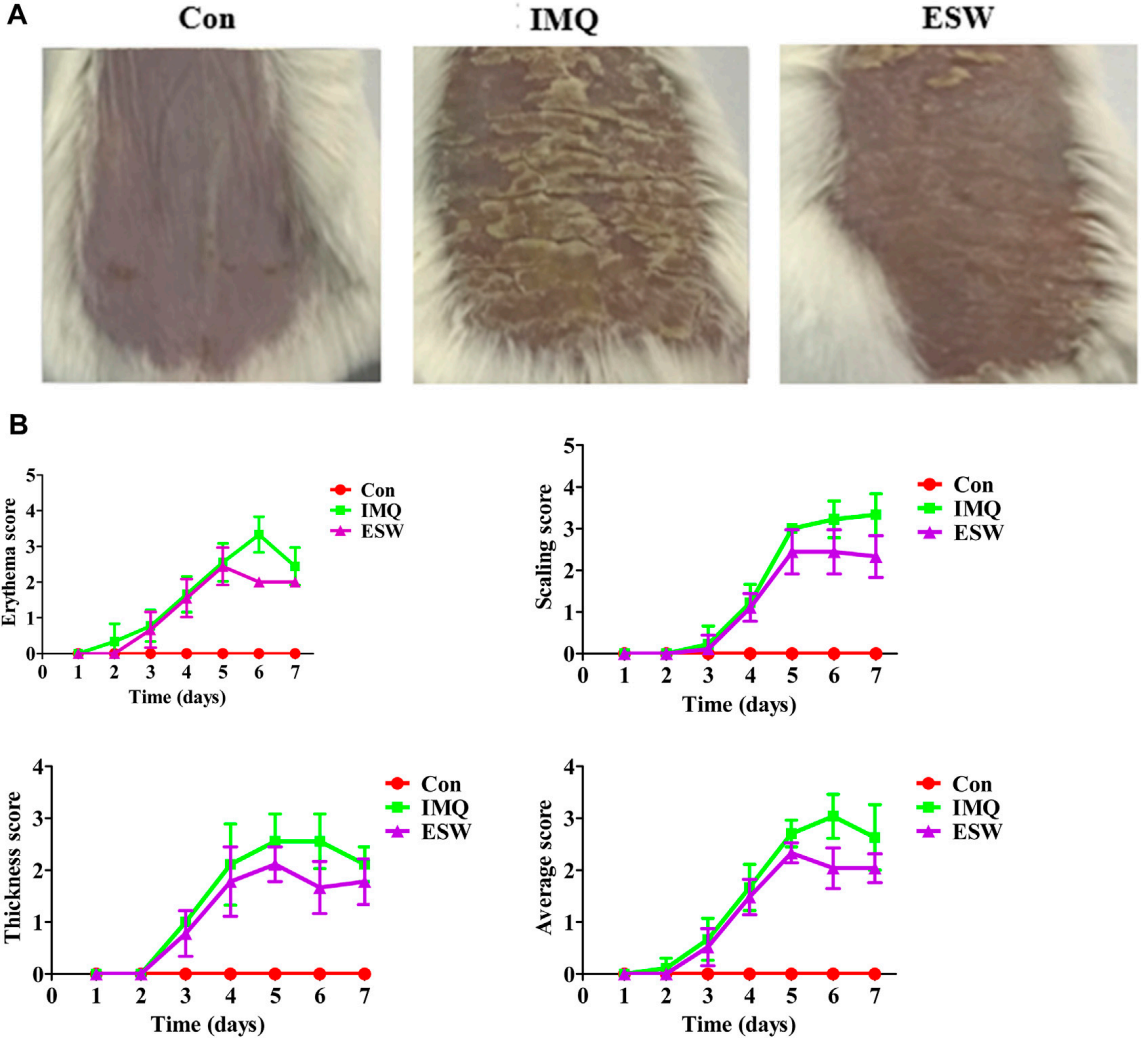
ESW alleviated the severity of IMQ-induced psoriasis-like skin inflammation. **(A)** Representative photos of the lesional skin of mice from each group. **(B)** The PASI scores of erythema score, scaled score, thickness score, and average score in each group from day 1 to day 7. All data were expressed as mean ± SD (*n* = 8).

Figure 2 showed the activity of oral intervention of ESW on PASI grading on IMQ-induced psoriasis mice. Photodocumentation confirmed the distinct IMQ-induced psoriasis phenotype and antipsoriatic effect of ESW (Figure 2A). Two or three days after the beginning of IMQ application, the IMQ group showed the development of obvious psoriasis phenotype. A typical example was shown in Figure 2B. From day third to day sixth, the PASI scores of erythema, scaling, and thickening of the skin continued to increase in the IMQ group. After consecutive several days of ESW intervention, inflammation was continually decreased in severity up to the last day of the experiment from days 5–7 onward, compared to those in the IMQ group, and the average PASI score was notably attenuated on the sixth day.

**FIGURE 3:**
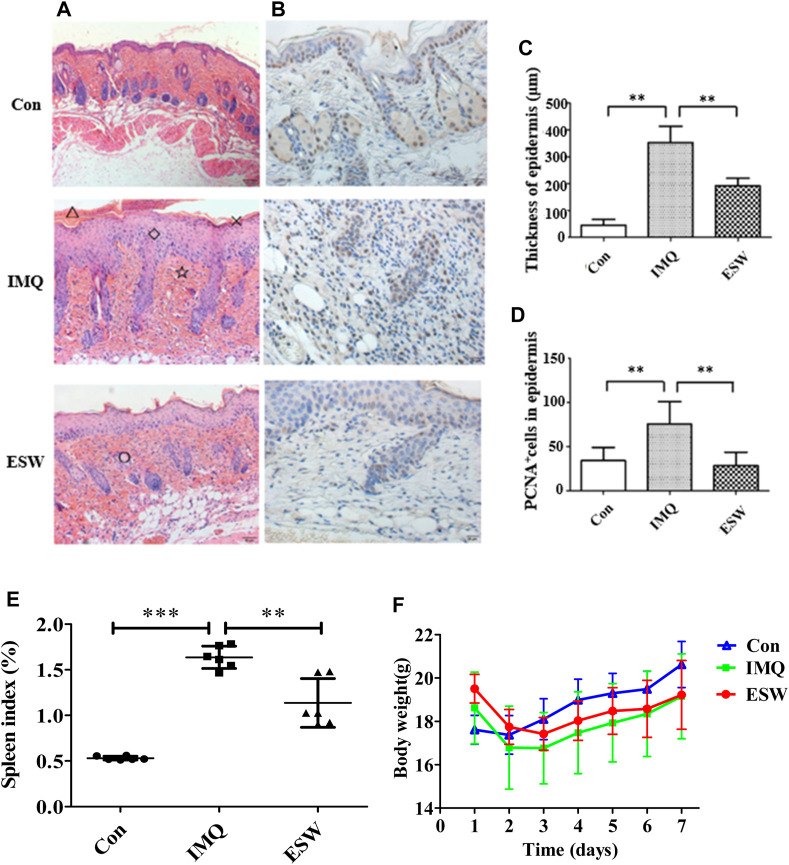
**(A)** Histological evaluation of skin lesions (H&E staining, magnification ×200); △: microlimb swelling; ×: parakeratosis; ◇: acanthosis; ☆: lymphocyte infiltration; ○: hair follicles and sebaceous glands. **(B)** Immunohistochemical staining (×200) for proliferating cell nuclear antigen (PCNA) (brown) in mouse back skin. **(C**,**D)** Thickness of epidermal and PCNA^+^ cells of the skin sections of mice from each group on day 7. **(E)** Histogram of the spleen index of each group. **(F)** Body weight change in each group from day 1 to day 7. Results were shown as the mean ± SD. **p < 0.01 or ***p < 0.001 vs. IMQ group.

Given the gross appearance observed using light microscopy (Figure 3), compared to the blank group, HE staining the skin of the group induced with IMQ showed severe parakeratosis, epidermal hyperplasias, accompanied by lymphocyte infiltration, and less marked parakeratosis, thickened epidermis, and cellular infiltration displayed in mice intervened with ESW comparing with IMQ group (Figure 3A). Moreover, compared with control mice, the epidermis thickness of the model group was remarkably enhanced after intervening with 5% IMQ (*p* < 0.01). Surprisingly, ESW treatment observably declined the epidermis thickness (*p* < 0.01).

Proliferating cell nuclear antigen (PCNA) is closely associated with cell repair and synthesis of eukaryotic cells. As such, it can be employed as a signal for measurement of the level of cell excessive proliferation (Celis and Celis, 1985), which is related to the pathogenesis of psoriasis. IHC staining of PCNA uncovered the positive expression rate of PCNA in the IMQ group, and the phenomenon was significantly reversed after treatment of ESW (*p* < 0.01) (Figures 3B,D). Compared with the Con group, the level of body weight in IMQ and ESW groups showed a significant reduction, but with less body weight in the IMQ group (Figure 3F). Furthermore, ESW significantly alleviated the spleen index (*p* < 0.01) (Figure 3E), which indicated that ESW might improve the pathological damage.

### Metabolomics Study

#### Multivariate Data Analysis of the Con, IMQ, and ESW Groups

**FIGURE 4:**
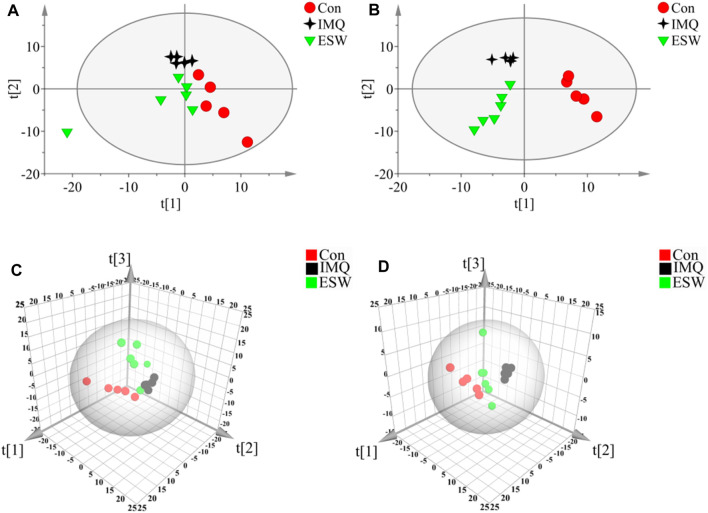
PCA and PLS-DA scores plots of mouse spleen data in Con group, IMQ group, and ESW group. **(A)** Dynamic mean-centered PCA 2D score plot of mouse spleen data. **(B)** Dynamic mean-centered PLS-DA 2D score plot of mouse spleen data. **(C)** Dynamic mean-centered PCA 3D score plot of mouse spleen data. **(D)** Dynamic mean-centered PLS-DA 3D score plot of mouse spleen data.

**FIGURE 5:**
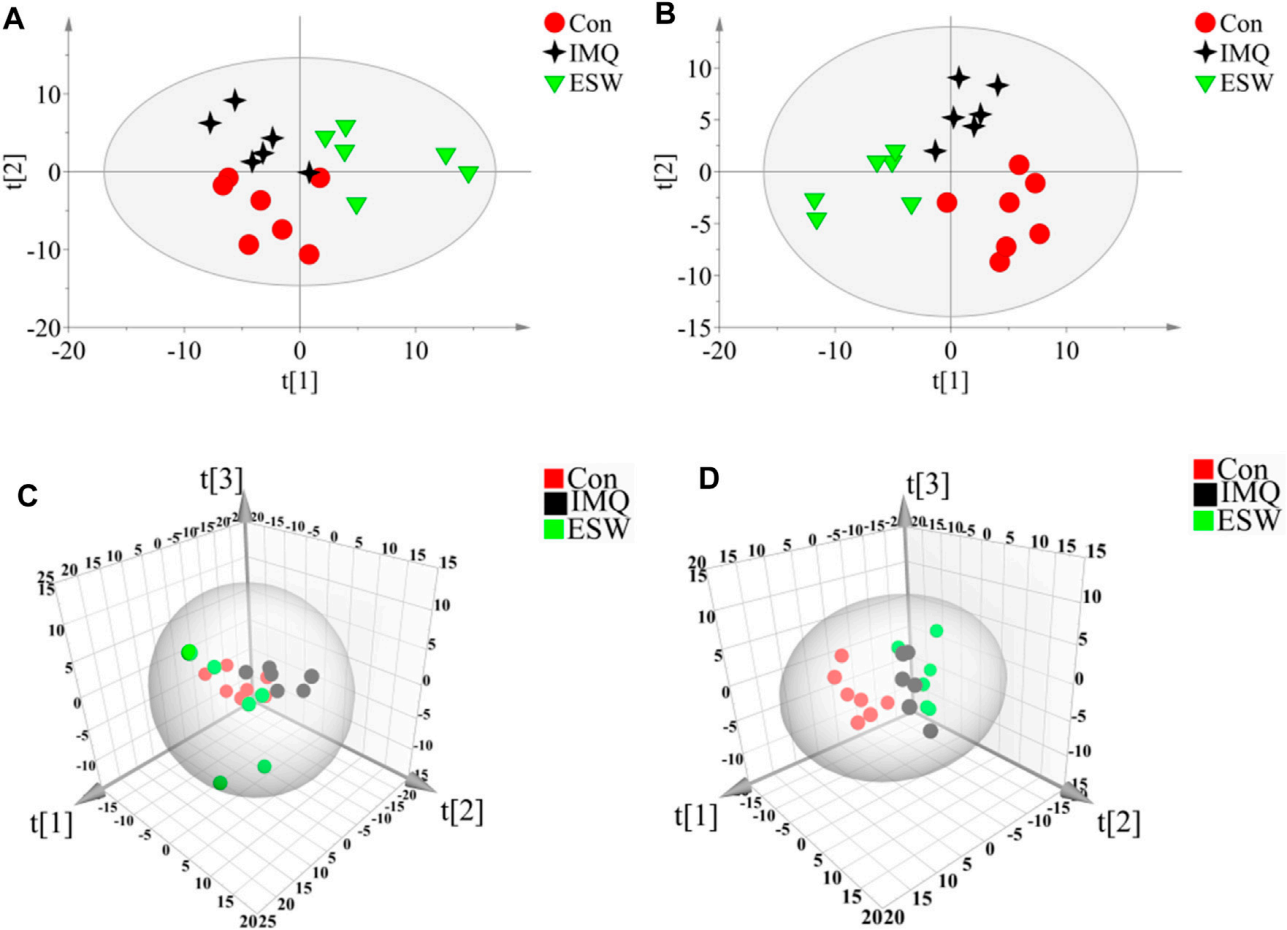
PCA and PLS-DA scores plots of mouse serum data in Con group, IMQ group, and ESW group. **(A)** Dynamic mean-centered PCA 2D score plot of mouse serum data. **(B)** Dynamic mean-centered PLS-DA 2D score plot of mouse serum data. **(C)** Dynamic mean-centered PCA 3D score plot of mouse serum data. **(D)** Dynamic mean-centered PLS-DA 3D score plot of mouse serum data.

**FIGURE 6:**
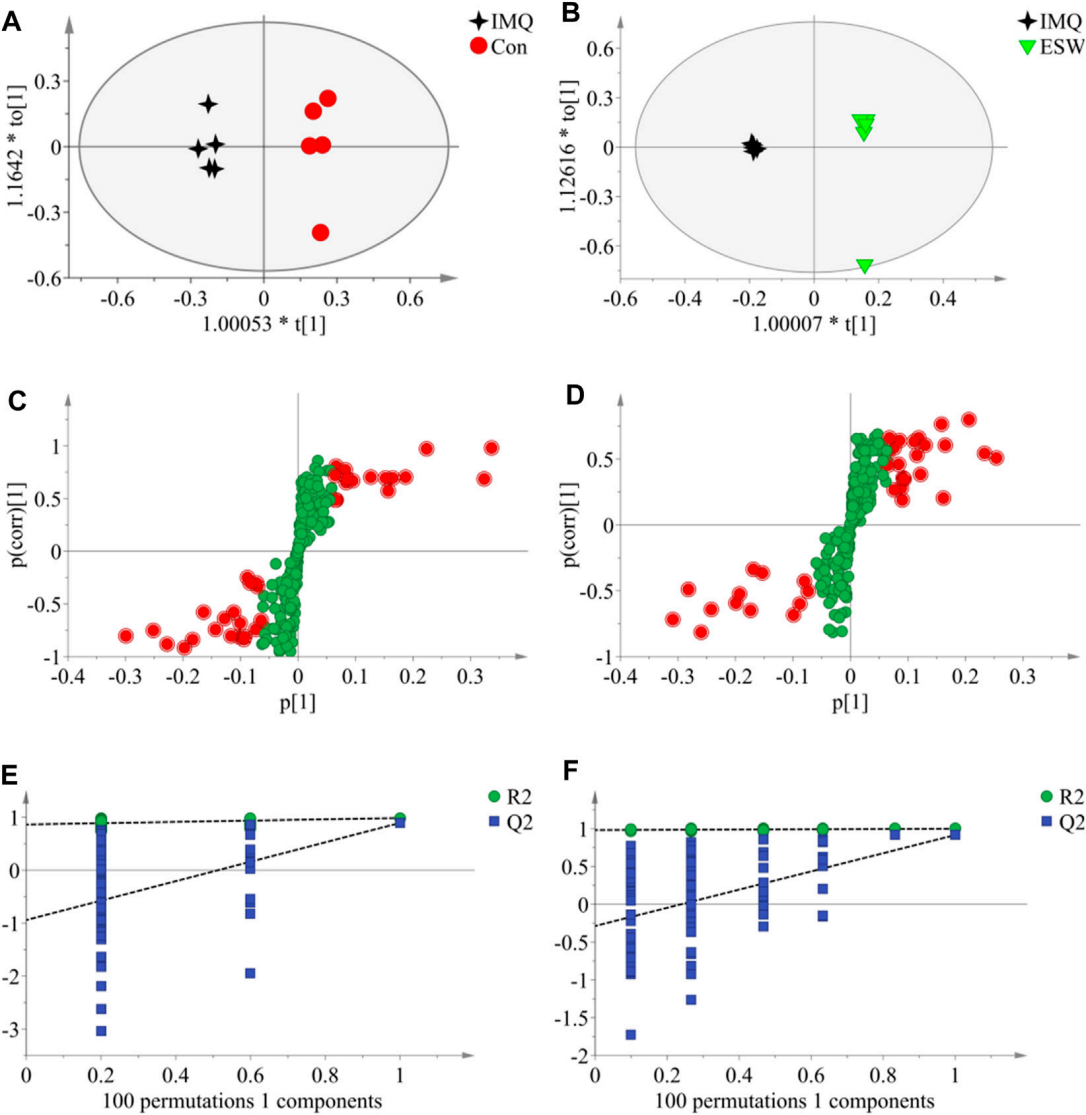
The OPLS-DA data of the spleen tissue. **(A**,**B)** OPLS-DA score plots comparisons between the Con and IMQ groups as well as the IMQ and ESW groups, respectively. **(C**,**D)** S-plots of the OPLS-DA model for the Con and IMQ groups as well as for the IMQ and ESW groups, respectively. **(E**,**F)** The 100-permutation test of the OPLS-DA model was for the Con and IMQ groups as well as for the IMQ and ESW groups, respectively.

**FIGURE 7:**
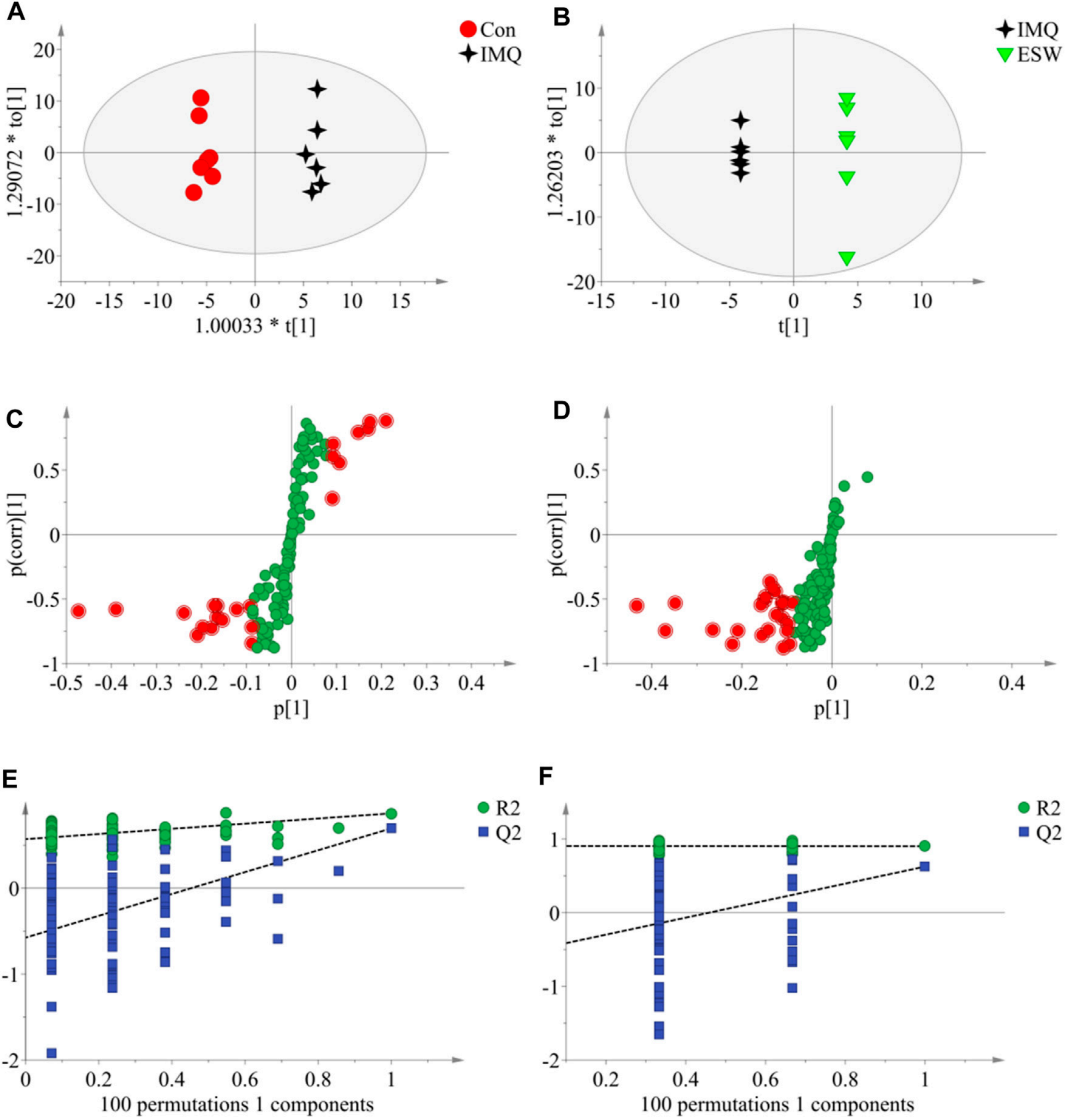
The OPLS-DA data of the serum. **(A**,**B)** OPLS-DA score plots comparisons between the Con and IMQ groups as well as the IMQ and ESW groups. **(C**,**D)** S-plots of the OPLS-DA model for the Con and IMQ groups as well as for the IMQ and ESW groups. **(E**,**F)** The 100-permutation test of the OPLS-DA model was for the Con and IMQ groups as well as for the IMQ and ESW groups.

In this research, a visualized multivariate analysis data result of spleen tissue and serum samples from the Con, IMQ, and ESW groups was shown in Figures 4, 5, respectively. In order to acquire information on classification and identify the metabolites, PCA and PLS-DA, two pattern recognition methods, were analyzed by SIMCA 14.1 software. PCA as an unsupervised statistical approach was applied to differentiate the discrepancy among the observations in the spleen and serum metabolomes. The results demonstrated that observed clusters exhibited a moderate difference in all groups (Figures 4A,C, 5A,C). PLS-DA, a supervised statistical approach, was employed for further strengthening the classification performance and showed an outstanding separation among three groups (Figures 4B,D, 5B,D). Moreover, further statistical classification of differences between the IMQ and Con or ESW groups in the metabolites was accomplished by OPLS-DA (Figures 6, 7). In the OPLS-DA model, there was an obvious difference between the IMQ and Con or ESW groups, which revealed a clear phenomenon of metabolic imbalance in organisms in the IMQ and ESW groups. Finally, 100 iterations-permutation experiments and the goodness of fit of the permutation test models were displayed to substantiate the model (Figures 6E,F, 7E,F). Based on the above results, it is illustrated that the disturbance of biomarkers appeared sequentially along with the pathological presentation of the mice in the IMQ group and the treatment improvement of the mice in the ESW group.

#### Potential Metabolites Identification in ESW Treatment

The VIP value beyond 1.0 and *p* value within 0.05 were considered to be the screening standards for the promising metabolites’ selection, which could identify the highlighted variables. Hence, all differential metabolites were selected as the variables based on VIP >1.0 and *p* < 0.05 in spleen homogenates and plasmas. It is worth noting that 17 potential candidates in spleen tissue (Table 2) and six potential candidates in serum (Table 3) were identified.

**TABLE 2:** Summary of potential metabolites in the spleen.

No.	Metabolites	Formula	Mass (m/z)	Retention time (min)	Fold change^a^		*p* value		VIP value^b^	
					Con vs. IMQ	IMQ vs. ESW	Con vs. IMQ	IMQ vs. ESW	Con vs. IMQ	IMQ vs. ESW
1	Urea^c^	CH_4_N_2_O	132.10	6.61	0.75	1.27	0.00	0.13	1.14	1.15
2	Glycine^c^	C_2_H_5_NO_2_	174.20	6.72	0.85	0.76	0.02	0.09	2.24	3.65
3	2-Butenedioic acid^c^	C_4_H_4_O_4_	204.20	7.91	1.14	1.00	0.03	1.00	2.57	0.02
4	L-Threonine^c^	C_4_H_9_NO_3_	291.28	8.23	0.87	1.03	0.04	0.76	1.01	0.24
5	Fructose^c^	C_6_H_12_O_6_	326.20	11.86	3.07	0.43	0.03	0.17	1.49	1.32
6	Heptanoic acid^c^	C_7_H_14_O_2_	73.12	14.44	1.27	0.92	0.01	0.38	1.29	0.58
7	L-Ascorbic acid^d^	C_6_H_8_O_6_	72.20	16.24	0.23	2.38	0.00	0.02	1.46	1.56
8	Quinoline^c^	C_9_H_7_N	305.25	18.29	1.53	0.94	0.00	0.30	5.26	1.47
9	D-Glucose^d^	C_6_H_12_O_6_	204.20	16.48	2.25	2.38	0.02	0.02	2.93	1.56
10	L-Proline^d^	C_5_H_9_NO_2_	142.20	7.16	0.87	0.80	0.04	0.01	1.99	2.49
11	Pyrimidine^d^	C_4_H_4_N_2_	241.20	7.66	0.69	1.85	0.01	0.00	1.48	4.07
12	Phosphoric acid^d^	H_3_PO_4_	357.2	13.14	0.34	1.69	0.00	0.03	3.10	2.73
13	L-Valine^e^	C_5_H_11_NO_2_	144.20	6.05	1.06	0.86	0.45	0.05	0.83	2.59
14	Malic acid^e^	C_4_H_6_O_5_	232.20	9.95	0.92	1.15	0.07	0.01	2.56	4.82
15	L-Phenylalanine^e^	C_9_H_11_NO_2_	192.20	11.33	1.36	0.67	0.17	0.03	1.03	1.85
16	d-Mannose^e^	C_6_H_12_O_6_	179.20	15.12	1.02	0.78	0.82	0.00	0.23	3.22
17	Z,Z-9,12-Octadecadienoic acid^e^	C_18_H_32_O_2_	253.20	20.35	3.71	0.23	0.17	0.03	0.76	1.34

a Fold change with a value >1 indicated a relatively higher intensity, whereas a value <1 indicated a relatively lower intensity.

b VIP value was obtained from the OPLS-DA model.

c Variables were obtained by comparing Con vs. IMQ (VIP >1 and *p* < 0.05).

d Variables were obtained by comparing IMQ vs. Con and ESW (VIP >1 and *p* < 0.05).

e Variables were obtained by comparing IMQ vs. ESW (VIP >1 and *p* < 0.05).

**TABLE 3:** Summary of potential metabolites in the serum.

No	Metabolites	Formula	Retention time (min)	Mass (m/z)	Fold change^a^		*p* value		VIP value^b^	
					Con vs. IMQ	IMQ vs. ESW	Con vs. IMQ	IMQ vs. ESW	Con vs. IMQ	IMQ vs. ESW
1	Myo-inositol^c^	C_6_H_12_O_6_	29.16	313.39	1.43	1.27	0.01	0.01	1.95	1.15
2	Z,Z-9,12-Octadecadienoic acid^d^	C_18_H_32_O_2_	32.23	339.40	1.62	1.28	0.02	0.47	1.06	0.26
3	Cholesterol^d^	C_27_H_46_O	42.00	129.10	1.61	0.99	0.00	0.97	2.01	0.12
4	d-Galactose^c^	C_6_H_12_O_6_	25.97	179.17	0.46	6.94	0.02	0.00	1.01	1.24
5	d-Mannose^e^	C_6_H_12_O_6_	26.20	321.30	1.15	1.82	0.88	0.02	0.01	1.62
6	D-Glucose^e^	C_6_H_12_O_6_	28.76	204.20	0.40	3.33	0.03	0.03	0.99	1.12

a Fold change with a value >1 indicated a relatively higher intensity, whereas a value <1 indicated a relatively lower intensity.

b VIP value was obtained from the OPLS-DA model.

c Variables were obtained by comparing IMQ vs. Con and ESW (VIP >1 and *p* < 0.05).

d Variables were obtained by comparing Con vs. IMQ (VIP >1 and *p* < 0.05).

e Variables were obtained by comparing IMQ vs. ESW (VIP >1 and *p* < 0.05).

**FIGURE 8:**
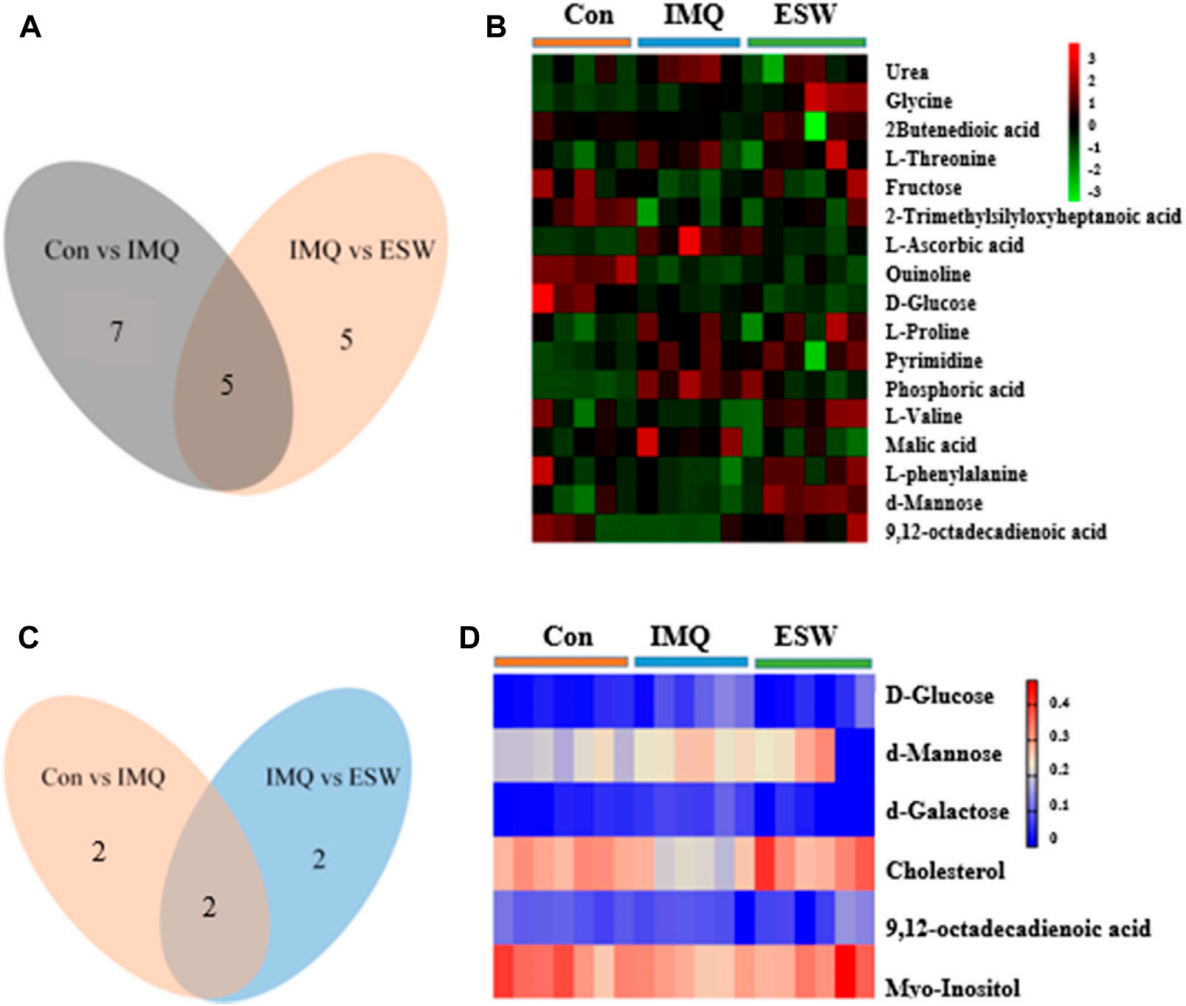
Venn diagram of the significant differential metabolites and heatmap of differential metabolites in the spleen **(A**,**B)** and serum **(C**,**D)**.

Compared with the Con group, the levels of urea, glycine, L-threonine, L-ascorbic acid, L-proline, pyrimidine, and phosphoric acid showed a significant increasing trend in the spleen of the IMQ group. In contrast, 2-butenedioic acid, fructose, heptanoic acid, quinolone, and D-glucose showed a marked decrease trend in the spleen of the IMQ group. Strikingly, also in the spleen tissue, ESW treatment led to the fall in contents of L-ascorbic acid, D-glucose, pyrimidine, phosphoric acid, and malic acid, as well as the rise in contents of L-proline, L-valine, L-phenylalanine, d-mannose, and Z,Z-9,12-octadecadienoic acid. Correspondingly, the inducement of IMQ resulted in the plasma dysfunction biomarkers including the lower levels of myo-inositol, Z,Z-9,12-octadecadienoic acid, and cholesterol, as well as d-galactose at a higher level. However, d-galactose, d-mannose, D-glucose, and myo-inositol were downregulated following ESW administration in plasma samples. The differential metabolites in psoriasis mice with ESW intervention were visualized by heatmap (Figure 8). Also interestingly, the Venn diagram (Figures 8A,C) showed a total of five coaltered metabolites among three groups in the splenic tissue and only two coaltered metabolites among three groups in the serum sample.

#### Biological Pathway Involved in Disease and Therapeutic Process

In the light of the pattern recognition analysis mentioned hereinbefore, the significant metabolites were labeled as the metabolic biomarkers in the Con, IMQ, and ESW groups. After that, we executed metabolic pathway analysis using the online tool MetaboAnalyst 3.0 and Human Metabolome Database to evaluate the significant variations among different groups (Tianjiao et al., 2014).

**FIGURE 9:**
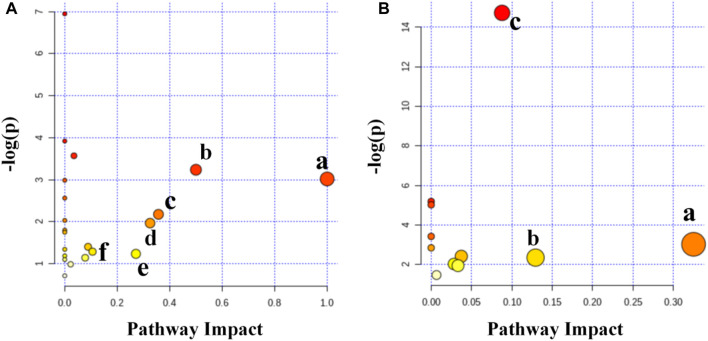
**(A)** Summary of pathway analysis in the spleen. **(a)** Linoleic acid metabolism, **(b)** phenylalanine, tyrosine, and tryptophan biosynthesis, **(c)** phenylalanine metabolism, **(d)** starch and sucrose metabolism, **(e)** glycine, serine and threonine metabolism, and **(f)** glyoxylate and dicarboxylate metabolism. **(B)** Summary of pathway analysis in the serum. **(a)** Starch and sucrose metabolism, **(b)** inositol phosphate metabolism, and **(c)** galactose metabolism.

Six primary altered pathways were identified in the spleen (Figure 9A) as follows: in the IMQ vs. Con/ESW groups, glyoxylate and dicarboxylate metabolism; glycine, serine, and threonine metabolism; linoleic acid metabolism; phenylalanine, tyrosine, and tryptophan biosynthesis; starch and sucrose metabolism; and phenylalanine metabolism were distinguished. Three primary altered pathways were identified in serum (Figure 9B), as follows: in the IMQ vs. Con/ESW groups, starch and sucrose metabolism; galactose metabolism; and inositol phosphate metabolism were distinguished.

## Discussion

*P. clypearia* and its Chinese patented drugs have been used for many years to treat a wide variety of inflammatory diseases. However, there are no reports about the pharmacological activities and metabolomics of *P. clypearia* on the psoriasis mice model. In the present study, we clarified that the ameliorating effect of ESW from *P. clypearia* in treating IMQ-induced psoriasis mice. Simultaneously, we offered a meaningful understanding of the underlying mechanism for ESW on psoriasis mice through a systemic technique of metabolomics.

The results of pathological observation and histological analysis showed a distinct recovery from psoriasis mice in the ESW treatment group. ESW significantly improved both individual and cumulative PASI scores, indicating that ESW could extenuate the skin symptoms including redness, scaling, and thickening. There was a gradual improvement in the body weight and spleen index of mice, as well as the low expression of PCNA in the ESW group.

Eventually, the metabolomics approach with multivariate analysis was employed for the sake of confirming differential metabolites and revealing perturbed metabolic pathways. Besides, identification of differential biomarkers and clarification of metabolic pathways could be an effective method for better comprehension of disease pathogenesis and drugs mechanism.

We investigated the metabolic pattern of psoriasis induced by IMQ and therapeutic influence of ESW using GC/MS combined with pattern recognition. A total of 17 potential biomarkers were identified in spleen samples. Compared to the Con group, the levels of urea, glycine, L-threonine, L-ascorbic acid, L-proline, pyrimidine, and phosphoric acid were increased significantly and the levels of quinoline, fructose, heptanoic acid, D-glucose, and 2-butenedioic acid were decreased observably after the intervention of IMQ, and three of these disorders could be reversed by ESW treatment. Psoriasis mice supplemented with ESW significantly lessened L-ascorbic acid, D-glucose, pyrimidine, phosphoric acid, and malic acid levels in the spleen organ accompanied by an increase in the contents of L-proline, L-valine, L-phenylalanine, d-mannose, and 9, 12-octadecadienoic acid (Table 2). Six potential biomarkers were changed dramatically in serum samples, of which the levels of myo-inositol, 9,12-octadecadienoic acid, and cholesterol were significantly reduced and the level of d-galactose was increased in the IMQ group compared to the Con group. After supplementing with ESW, it was also accompanied by reduction of d-galactose, d-mannose, D-glucose, and myo-inositol levels. Consequently, it could be inferred that the mechanism underlying the treatment of ESW on psoriasis was closely related to these obviously changed metabolites.

In order to clarify the biochemical and histological effects of ESW in metabolomic profiling taking a step forward, the potential biomarkers of the two sets of comparisons were inputted to the online system, MetaboAnalyst (Figure 9). ESW was proved to influence the metabolic pattern of the psoriasis model. The ESW-treated group displayed an inclination of recovering to a normal condition. Notably, every metabolic pathway above two set thresholds (0.1 or 0.05) was categorized as a promising targeted pathway. The main six metabolic pathways in spleen consisted of linoleic acid metabolism, starch and sucrose metabolism, glyoxylate and dicarboxylate metabolism, phenylalanine metabolism, glycine, serine, and threonine metabolism, and phenylalanine, tyrosine, and tryptophan biosynthesis. The top three metabolic pathways in serum were galactose metabolism, starch, and sucrose metabolism and inositol phosphate metabolism.

Metabolites that play significant effects on the related pathway have a primary role in the pathogenesis and potential complications of the disease (Tsoukalas et al., 2019). It has been reported that incremental urea at concentrations is recognized as one of the major causes of the disintegration of the gut epithelial dysfunction, vascular smooth muscle cell apoptosis, endothelial barrier, oxidative stress, and disorder in adipocytes, thus directly leading to transferring of bacterial toxins into the bloodstream and systemic inflammation, cardiovascular disease, and insulin resistance (Lau and Vaziri, 2017). In our study, an elevated level of urea was also observed in mice with psoriasis, similar as described previously (Kang et al., 2017). In addition, the production of urea was closely related to the urea cycle. The change activity of the urea cycle and lacking balance between arginine partitioning to urea versus NO synthesis was not the only one to inflammation and might be a common metabolic characteristic of cellular stress that could be resisted by pyruvate carboxylase-mediated ureagenesis. Moreover, the connection of mitochondrial pyruvate handling and arginine utilization was able to disclose an anti-inflammatory action of glucose metabolism through regulating the urea cycle (Fu et al., 2020). Our findings that there were disturbing levels of glucose and urea during the experiment explained that the changes in small molecule metabolites were linked to the disease outcomes in these settings. Psoriasis is believed to be correlated with numerous cardiovascular risk factors such as abnormal cholesterol level (Choudhary et al., 2020). There was a related report that the application of IMQ resulted in a diet and weight loss and subsequently lipid levels reduction, particularly TC and LDL in mice (Xie et al., 2017). In a randomized placebo-controlled trial of Secukinumab on aortic vascular inflammation in moderate-to-severe plaque psoriasis (VIP-S), the level of cholesterol was increased lightly after treatment, which was consistent with our research results (Gelfand et al., 2020). Compared to normal skin, tissue biopsies from psoriatic skin involved a lower level of the metabolites myo-inositol (Sitter et al., 2013). Accordant with preceding research, the significant changes of myo-inositol were displayed in the IMQ group and ESW group of our study, which might due to the disorder of myo-inositol metabolism caused by the application of IMQ and subsequently altered inositol phosphate metabolism in serum. Glycine, as a regulator of the inflammatory cascade, had a wide range of biological activities and might be a useful supplement for individuals with inflammation of the Achilles tendon (Vieira et al., 2015). Glycine can improve the viability and counteracts deleterious responses to LPS/IFN-γ in BV-2 microglial cells, which might be relevant in neurodegenerative processes associated with inflammation (Egger et al., 2020). Furthermore, the supplementation of N-acetyl cysteine and glycine could ameliorate diastolic function in the aged mouse and might have the ability to prevent important morbidities for the elderly (Cieslik et al., 2018). Psoriasis, as evidence, has accumulated all these years, usually is associated with cardiovascular disease, and leads to a higher prevalence and death rate of a cardiovascular event (Kim et al., 2018; Mehta et al., 2018; von Stebut et al., 2019). In this work, the level of glycine increased in the IMQ group, indicating that, due to the self-healing function of the body, the mouse treated with IMQ could produce more glycine to prevent the further development of psoriasis. Additionally, a higher level of metabolite assessment from pathway to network can be applied to understand the physiological characteristics of metabolites that influenced the biological status. Glyoxylate and dicarboxylate metabolism pathway, a primary pathway for the enrichment of the amino acid glycine, was also reported significantly disturbed in patients with systemic lupus erythematosus which is an autoimmune disease resembling psoriasis (Gunawan et al., 2018; Zhang et al., 2019). Likewise, studies have shown that the disturbed metabolic networks centered on glycine and arachidonic acid, including linoleic acid metabolism, glyoxylate, and dicarboxylate metabolism and glycine, serine, and threonine metabolism, might be involved in the significant inhibition of systemic inflammatory responses (Yao et al., 2015). Thus, we proposed that the occurrence of psoriasis may be related to the regulation of glycine-involved metabolic pathways. In the research process of psoriasis, the difference of 2-butenedioic acid and quinoline compared with controls was first noticed, and it was speculated that the pathogenesis of psoriasis might be related to the changes in 2-butenedioic acid and quinoline levels. On the other hand, some monosaccharides were absorbed and metabolized in various ways. Particularly, fructose was metabolized different from glucose, and its metabolism was able to cause notable inflammation and visceral adiposity whereas excessive glucose intake mainly contributed to the augmentation of subcutaneous fat (Stanhope et al., 2009; Tchernof and Després, 2013). This provided a reasonable explanation for the weight loss of mice with inflammatory skin disease and the decrease of glucose and fructose levels in the spleen in this study. The perturbation of fructose and glucose might also cause the occurrence of psoriasis by raising the need for protein biosynthesis and keratinocyte hyperproliferation (Kang et al., 2017; Makuch et al., 2020). To the best of our knowledge, abnormal energy metabolism is ubiquitous in animal models of TH17 cell-mediated autoimmune diseases (Shen and Shi, 2019). In the IMQ group, glucose concentration was significantly reduced in spleen (Table 2), interfering with starch and sucrose pathway, which might subsequently lead to energy metabolism disorder and the occurrence of psoriasis. As compared to the Con group, the concentrations of fructose, glucose, myo-inositol, and cholesterol were decreased, while those of urea and glycine were increased in the IMQ-induced psoriasis group. Serving as the intermediate substance, the differential metabolites are involved in glyoxylate and dicarboxylate metabolism, glycine, serine and threonine metabolism, starch and sucrose metabolism, and inositol phosphate metabolism.

dl-Malic acid which usually presented in the form of α-hydroxy acids was applied as an exfoliating agent. The research has shown that the high-dose malic acid group could not only effectively improve atopic dermatitis induced by 2,4-dinitrochlorobenzene but also availably ameliorate inflammatory reaction in a human keratinocyte cell line. Properly speaking, it was able to restrain phosphorylation of MAPK and NF-κB in skin tissue, decrease the levels of interleukin-4 and IgE in serum, and reduce the expression level of various inflammation-related cytokines, thereby ameliorating the skin condition of atopic dermatitis (Lee et al., 2019). In this study, the malic acid level in the spleen of ESW-treated mice was significantly decreased compared to the IMQ group (Table 2). According to this result, it was speculated, that due to differences in disease models and detection organs, the effect of ESW on psoriasis might be inversely proportional to the level of malic acid. Agavins supplementation mitigated metabolic endotoxemia and low-grade inflammation in association with gut microbiota regulation and their metabolic products and l-valine was identified as possible biomarkers for this prebiotic supplement (Huazano-García et al., 2020). The differential diagnosis of seronegative rheumatoid arthritis (negRA) and psoriasis arthritis (PsA) is often difficult due to the similarity of symptoms and the unavailability of reliable clinical markers. A study found that valine could be used as a biomarker to discriminate against them (Souto-Carneiro et al., 2020). The outcome of the present study indicated that ESW treatment might be associated with an increased level of valine. The metabolism of aromatic amino acids such as tryptophan and phenylalanine, in especial inflammation-induced metabolic disorders, played an important action in regulating immune cell function. Psoriasis has a comprehensive psychosocial and emotional disturbance for the patients beyond the physical dimensions of disease, leading to humiliation, poor self-esteem, and incremental stress (Kim et al., 2017). The obvious mechanisms of psychiatric disorders like anxiety and major depressive disorders were closely related to the disturbance of neurotransmitter biochemistry. The important pathway of neurotransmitter biosynthesis mainly consisted of the adrenergic, noradrenergic, and dopaminergic pathways derived from the usual intermediate L-3,4-dihydroxyphenylalanine (L-DOPA). And most importantly of all, the production of L-DOPA relied on the precursor molecules phenylalanine and tyrosine (Strasser et al., 2017). Phenylalanine contributed to phenylalanine metabolism and the biosynthesis of phenylalanine, tyrosine, and tryptophan in ESW-improved psoriasis mice. In our study, phenylalanine level in ESW-improved psoriasis mice was increased compared to IMQ mice. It can be speculated that ESW may improve the state of mice with psoriasis by changing biochemical substances which was closely bound up with emotions. Except for amino acids and sugars overexpression, fatty acid expression displayed an increase in the spleen of ESW treatment mice. The existing researches clearly showed that the application of fatty acid could decrease cardiovascular risk factors and give assistance to the treatment of chronic inflammatory diseases (Baril-Gravel et al., 2015; Dawczynski et al., 2018; Markworth et al., 2018). Linoleic acid is an unsaturated fatty acid, effectively reversed the inflammatory responses induced by palmitic acid treatment in microglial cells (Tu et al., 2019). Simultaneously, dietary conjugated linoleic acid has modulation of inflammation and immunity (Viladomiu et al., 2016). Interestingly, the intervention of supplementation plus physical exercise caused a significant decrease of linoleic acid and omega-6 polyunsaturated fatty acids in sarcopenic elderly patients (Corsetto et al., 2019). In the ESW group, 9,12-octadecadienoic acid, which was an important source of linoleic acid metabolism for animals, increased significantly compared to the model group.

Based on the above, the improvement of the psoriasis phenotype after ESW administration may be bound up with the metabolism of the above amino acids, sugars, and fatty acids. And compared with serum samples, there are more differential metabolites and metabolic pathways changed in spleen samples, suggesting that the spleen may play an important role in inflammatory diseases (Barrea et al., 2018).

## Conclusion

In the current paper, oral administration of 2.4 g/kg ESW for six consecutive days for effective treatment of psoriasis was visualized by apparent characteristics and histology. In view of high-throughput and high-resolution metabolomics analysis method of GC/MSD united with cluster analysis and metabolic pathway analysis, it was discovered that ESW could ameliorate psoriatic lesions by acting on 17 different biomarkers in spleen samples which regulated 21 metabolic pathways and six diverse variances in serum samples which regulated 11 metabolic pathways. The most critical pathway in spleen was linoleic acid metabolism, phenylalanine metabolism, glyoxylate and dicarboxylate metabolism, glycine, serine and threonine metabolism, starch, and sucrose metabolism, and phenylalanine, tyrosine, and tryptophan biosynthesis. Additionally, three significant metabolic pathways were enriched in serum samples such as starch and sucrose metabolism, inositol phosphate metabolism, and galactose metabolism.

Our study reported for the first time that a commonly used traditional Chinese medicine, *P. clypearia* has a therapeutic effect on psoriasis and its mechanism in regulating metabolism. The six ingredients identified by the UHPLC-MS/MS method may be the key pharmacological components of ESW, providing a direction for the further exploration of the active monomer compounds and molecular mechanism of the treatment of *P. clypearia* on psoriasis. Moreover, the plant resources of *P. clypearia* will be more fully utilized based on its potential value in the treatment of psoriasis. However, due to the restricted time and the limited economic, we investigated only spleen and serum samples and did not investigate dynamic changes in metabolomics profiles over time. Therefore, further researches will pay close attention to gather different tissue samples and illuminate the dynamic metabolomic profile for the sake of elucidating the mechanism of action of *P. clypearia*.

## Data Availability

The raw data supporting the conclusions of this article will be made available by the authors, without undue reservation.

## Abbreviations

ACN, acetonitrile; BSTFA, bis(trimethylsilyl)trifluoroacetamide; CAD, Collision Gas; CUR, Curtain Gas; ESW, ethanol fraction of water extract; GC-MS, gas chromatography-mass spectrometer; GS1, Ion Source Gas 1; GS2, Ion Source Gas 2; H&E, Hematoxylin and Eosin; IL-23, interleukin-23; IMQ, imiquimod; IS, IonSpray Voltage; LC-MS, liquid chromatography-mass spectrometry; L-DOPA, L-3,4-dihydroxyphenylalanine; MRM, multiple reaction monitoring; NMR, nuclear magnetic resonance; OPLS-DA, orthogonal partial least-squares discriminate analysis; PASI, psoriasis area and severity index; PCA, principal components analysis; *P. clypearia*, *Pithecellobium clypearia* Benth.; PCNA, proliferating cell nuclear antigen; PLS-DA, partial least-squares discriminant analysis; QC, quality control; TCM, traditional Chinese medicine; TIC, total ion current chromatogram; TIS, turbo ion spray; TNF-α, tumor necrosis factor-α; UHPLC-MS/MS, ultrahigh-performance liquid chromatography combined with tandem mass spectrometry; VIP, variable importance in the projection value.
